# Dr. Fawssett
*The following has been sent to us by a valued Correspondent.


**Published:** 1823-11

**Authors:** 


### DR. FAWSSETT.*

We have to announce the premature death of Dr. FAWSSETT, an eminent provincial physician. This event took place at Horncastle, in Lincolnshire, on the 11th of this month, after a long and painful indisposition, which he bore with the submission of a Christian. He was the eldest son of Richard Fawssett, Esq. of Holbeach, who lived to an advanced age, and exercised the different branches of the healing art with great success to a late period of his life. Dr. Fawssett received his classical education at Louth, and distinguished himself so much that he became a favourite pupil of the Rev. Mr. Emeris, formerly master of the grammar-school there. He obtained the rudiments of his medical education at the Horncastle Dispensary, which he attended with great diligence for four years. From thence he removed to the University of Edinburgh, where he succeeded to the degree of doctor of physic A.D. 1798. The thesis selected for his inaugural dissertation, in compliance with the established regulations of that pre-eminent school of medicine, was on the Cure of Intermittent Fevers. These diseases were at that time very prevalent in different parts of Lincolnshire. Dr. Fawssett has treated his subject in a very masterly manner, and recommended, from experience at the Dispensary, several indigenous substances, which are highly deserving of attention. Since the improved system of draining the low lands, intermittents have nearly disappeared in the Lincolnshire Fens, and the few that do show themselves are much milder, and of easy cure. Having attended the hospitals and lectures in London for another season, he was invited by his late preceptor, Dr. Harrison, to commence

* The following has been sent to us by a valued Correspondent.

his medical career at Horncastle, where he was a general favourite. Here he was actively employed, both in private and dispensary practice, as long as health would permit of his exertions. On Dr. Harrison's leaving Horncastle, Dr. Fawssett succeeded to all the employment of a very extensive district, in which he lived universally respected and esteemed.

Lately deceased, J. N. JOHNSON, M.D. Fellow of the Royal College of Physicians, &c.

### METEOROLOGICAL JOURNAL,

